# Podiatry intervention versus usual care to prevent falls in care homes: pilot randomised controlled trial (the PIRFECT study)

**DOI:** 10.1186/s12877-017-0541-1

**Published:** 2017-07-12

**Authors:** Gavin Wylie, Hylton B. Menz, Sarah McFarlane, Simon Ogston, Frank Sullivan, Brian Williams, Zoe Young, Jacqui Morris

**Affiliations:** 1 NHS Tayside, Ageing and Health, and School of Nursing & Health Sciences, University of Dundee, Dundee, UK; 2 School of Allied Health, College of Science, Health and Engineering, La Trobe University, Victoria, Australia; 3 NHS Tayside and Nursing & Midwifery Research Unit, Glasgow Caledonian University, Glasgow, UK; 4 School of Medicine, University of Dundee, Dundee, UK; 5 School of Medicine, University of St Andrews, St Andrews, UK; 6 School of Health and Social Care, Napier University, Edinburgh, UK; 7 NHS Tayside, Perth, UK; 8 School of Nursing and Health Sciences, University of Dundee, Dundee, UK

**Keywords:** Older people, Falls, Podiatry, Care homes

## Abstract

**Background:**

Common foot problems are independent risk factors for falls in older people. There is evidence that podiatry can prevent falls in community-dwelling populations. The feasibility of implementing a podiatry intervention and trial in the care home population is unknown. To inform a potential future definitive trial, we performed a pilot randomised controlled trial to assess: (i) the feasibility of a trial of a podiatry intervention to reduce care home falls, and (ii) the potential direction and magnitude of the effect of the intervention in terms of number of falls in care home residents.

**Methods:**

Informed by Medical Research Council guidance on developing and evaluating complex interventions, we conducted a single blind, pilot randomised controlled trial in six care homes in the East of Scotland. Participants were randomised to either: (i) a three month podiatry intervention comprising core podiatry care, foot and ankle exercises, orthoses and footwear provision or (ii) usual care. Falls-related outcomes (number of falls, time to first fall) and feasibility-related outcomes (recruitment, retention, adherence, data collection rates) were collected. Secondary outcomes included: generic health status, balance, mobility, falls efficacy, and ankle joint strength.

**Results:**

474 care home residents were screened. 43 (9.1%) participants were recruited: 23 to the intervention, 20 to control. Nine (21%) participants were lost to follow-up due to declining health or death. It was feasible to deliver the trial elements in the care home setting. 35% of participants completed the exercise programme. 48% reported using the orthoses ‘all or most of the time’. Completion rates of the outcome measures were between 93% and 100%. No adverse events were reported. At the nine month follow-up period, the intervention group per-person fall rate was 0.77 falls vs. 0.83 falls in the control group.

**Conclusions:**

A podiatry intervention to reduce falls can be delivered to care home residents within a pilot randomised controlled trial of the intervention. Although not powered to determine effectiveness, these preliminary data provide justification for a larger trial, incorporating a full process evaluation, to determine whether this intervention can significantly reduce falls in this high-risk population.

**Trial registration:**

ClinicalTrials.gov identifier: NCT02178527; Date of registration: 17 June 2014.

**Electronic supplementary material:**

The online version of this article (doi:10.1186/s12877-017-0541-1) contains supplementary material, which is available to authorized users.

## Background

Half of all care home residents fall every year, often several times [1–3]. They are at an increased risk of falls compared to community dwelling older people because they are more frail, and frequently have multiple long-term health problems [4], for which they are taking multiple medications known to impair balance [5]. Across community and care home dwelling older people, falls are one of the most common causes of hip fracture, unplanned hospitalisation and death [6]. Despite the disproportionately high number of falls in care homes, a Cochrane systematic review found the evidence supporting effectiveness of interventions to reduce falls in care homes to be equivocal [7]. One recent feasibility randomised controlled trial (RCT) has shown that a risk assessment intervention to reduce care home falls is implementable under trial conditions, but it has not yet been tested in a full scale RCT [8].

Falls result from complex interplay between environmental hazards and physiological risk factors such as impaired muscle strength, balance and gait [5]. Chronic disease that leads to impairments in sensory, neurological, cognitive and musculoskeletal functioning also increases the risk of falls [9]. More recently, foot problems, which are common in older people [10, 11], have been shown to be a contributing factor to falls [12, 13]. Foot and ankle risk factors for falls are: reduced ankle joint range of motion, hallux valgus deformity (bunion), decreased toe plantarflexor strength, increased shoe heel height, inadequate shoe fixation (absence of strap or lace or other retaining medium) and reduced shoe-sole contact area [12, 14, 15]. Furthermore, recent reviews suggest that foot and ankle exercises (particularly toe exercises), and footwear interventions (shoes or foot orthoses) may improve falls-risk related outcomes such as static and dynamic balance ability, ankle flexibility and lateral stability [16, 17].

Given the demonstrable link between feet, footwear and falls, an inexpensive and simple to implement multifaceted podiatry intervention has been developed and studied within an Australian community dwelling cohort of older people in a large RCT [18]. This work demonstrated that the intervention group experienced 36% fewer falls compared to those in the control group, and significantly reduced the incidence of falls compared to usual care (incidence rate ratio 0.64, 95%; CI 0.45 to 0.91). More recently, a cohort-RCT using a similar podiatry intervention conducted with a United Kingdom (UK) community dwelling population demonstrated a reduction in the incidence rate of falls in the intervention group (incidence rate ratio 0.88, 95% CI 0.73 to 1.05) and a reduction in the proportion of multiple fallers (27.6% vs 34.6%) [19]. The podiatry intervention holds promise as a falls-reduction strategy. However, the results of falls trials conducted in community dwelling older people cannot be directly applied to those living in care home settings who are more frail, prone to sudden declines in health, and more cognitively and functionally impaired than those living in their own homes. Therefore, exploring the feasibility and potential effectiveness of the intervention in the care home setting – the population most at risk of falls – is warranted.

Challenges in delivering interventions and conducting trials in care homes include recruitment, engaging care home staff, and ensuring that the intervention is feasible and deliverable [20, 21]. Given these uncertainties, we conducted a two-stage study that was informed by the Medical Research Council (MRC) guidance on complex intervention development [22] (the PIRFECT study – Podiatry Intervention to Reduce Falls in Elderly Care Trial). In the first stage we remodelled the existing multifaceted podiatry intervention that was designed for community-dwelling older people [18] to ensure that it was feasible and acceptable in the care home setting [23]. We also refined recruitment processes and determined appropriate outcomes [24]. The second stage of the study, which is reported here, was a pilot RCT of the remodelled multifaceted podiatry intervention.

We aimed to establish the feasibility of a future phase III multicentre RCT designed to establish the clinical effectiveness of the remodelled multifaceted podiatry intervention. The objectives of this pilot study were to examine the recruitment and retention rate, adherence, the quality of the outcome data, and to estimate the potential effect size of the intervention on the number of falls. We anticipated that this approach would inform decision-making regarding progression to a large-scale definitive phase III RCT.

## Methods

### Design

The study was a two arm, parallel group pilot randomised controlled trial with outcome assessments at baseline (T1), and subsequent blinded outcome assessment at the end of the intervention (T2) and at 3 months (T3) and 6 months (T4) after the end of the intervention.

### Setting

Six care homes in the East of Scotland. Four care homes were privately owned, one was operated by a charitable trust, and one was state-run by the local authority. The capacity of each care home ranged from 24 to 55 residents. Each of the privately owned care homes always had at least one registered nurse on duty, with the remaining staff being care assistants. The state-run and charitable trust care homes employed no registered nurse (although had regular visits from UK National Health Service nurses), and were staffed entirely by care assistants. Our initial approach to care homes was through a number of routes: directly to the care home, through regulators (the Scottish Care Inspectorate), and via UK National Health Service (NHS) falls service co-ordinators. We met with care home managers who expressed interest to explain in full what was involved in participating in the study. Those who wanted to take part then provided written permission to conduct the study within their homes. The care homes that were recruited were representative of normal UK care home provision and typical of care homes that would be invited to take part in a future multicentre RCT.

### Participants and recruitment

The East of Scotland Research Ethics Service granted ethical approval for the study (reference 12/ES/0088). Care home staff identified and initially approached residents who met the initial inclusion criteria on behalf of the research team. A member of the research team then approached interested residents, to provide further information and answer any questions. A researcher then screened the potential participant using the inclusion/exclusion criteria before obtaining written informed consent.

Participants were eligible for recruitment if they fulfilled the following inclusion criteria: (i) >65 years old and permanently living in a care home for older people; (ii) one or more falls in the previous year (in order to target those most at risk of a fall [25]); (iii) a foot problem within the scope of practice of a UK trained podiatrist (defined by the UK Health and Care Professions Council [26]). Residents were excluded if they were: (i) terminally unwell or too frail to be included; (ii) only able to mobilise with the use of wheelchair; (iii) temporary residents; (iv) unable to provide informed consent; (v) lower limb amputees.

We used the mini-mental-state exam (MMSE) [27] to guide our assessment of capacity to consent. We used a severe score (0–9) on the MMSE as an indicative threshold for inability to provide informed consent. However, the final decision to recruit, irrespective of MMSE score, was through assessment by the research podiatrist, based on the ability of the prospective participant to explain back to the research podiatrist what was required by their participation in the study. Where there was ambiguity, a decision on whether or not a potential participant should enter the study was based on agreement between two researchers (GW and ZY).

### Randomisation and blinding

After recruitment, but prior to randomisation, baseline assessments were completed by one of 2 research podiatrists (GW or ZY). Participants were then randomly assigned to the usual care control group or the intervention group. Randomisation was conducted via a concealed, web-based randomisation service provided by UK Clinical Research Collaboration (UKCRC) registered Tayside Clinical Trials Unit at the University of Dundee. Minimisation was employed to ensure balance between the two groups on the following falls-related risk factors: age, gender, presence of polypharmacy (>4 concurrent medications) and the presence of psychotropic medication. Follow up measures were conducted by a rater who was a physiotherapist of 20 years experience (SM), trained in the conduct of all measures and who was unaware of participant allocation. Given the nature of the intervention it was not possible to blind participants and care home staff to group allocation, however blinding of the rater was maintained by ensuring that access to research records indicating group allocation was restricted and by asking participants not to reveal their group allocation to the rater at the outset of each follow up visit.

### Outcome measures

Feasibility outcome data and falls-related outcome data were collected. Feasibility data were collected on recruitment, retention, adherence, and missing data. The primary falls outcome measure was the number of falls in the 9-month trial period. A fall was defined as ‘*an unexpected event in which the participant comes to rest on the ground, floor or lower level* [28]’. Falls recall by care home residents themselves is unreliable [29], therefore data on the number of falls were collected via the accident reporting systems in place within the care homes. All homes kept accident records that recorded both the number of falls and the circumstances surrounding them (where possible), which allowed us to count only falls meeting our definition. We also calculated the time to first fall to assess whether those in the intervention group increased their risk of falls as a result of the intervention. The secondary outcome measures were:

I. Podiatry Objective Clinical Score (POCS) [30]: a clinical measure of current foot problems determined by a podiatrist and scored from 1 (no problems) to 5 (gross problems);
II. Berg Balance Scale (BBS) [31]: a measure of balance function in older people and scored from 0 to 56 where a higher score indicates lower falls risk;
III. Timed Up and Go Test [32]: a simple assessment of mobility where the participant is timed to rise from a chair, walk 3 m, turn round, walk back to the chair and sit down where a time of 30 s or more to complete the manoeuvre infers that the person is at increased risk of falls;
IV. Barthel Index [33]: a measure of competence in activities of daily living and scored from 0 to 100 where a higher score indicates greater independence;
V. EQ-5D [34]: a measure of generic health status where a summary index score between zero and one is derived, with one representing the best possible health state;
VI. Nursing Home Falls Self-Efficacy Scale (NHFSS) [35]: a measure of falls efficacy where a higher score indicates less fear of falling
VII. Ankle joint muscle strength: measured via a dynamometer in Newtons [36] whereby a higher score indicates better muscle strength.

Falls outcome data were collected at the following time points: baseline (T1), immediately at the end of the 3-month intervention (T2), 3 months after the end of the intervention (T3), and finally 6 months after the end of the intervention (T4).

### Development of the intervention

The empirical basis for the intervention was a multifaceted podiatry intervention to reduce falls, previously evaluated in community dwelling Australian and UK populations [18, 19]. Prior to conducting the study described in the present paper, the intervention was delivered by the research podiatrist in an acceptability study to care home residents (*n* = 8) and care home staff (*n* = 5) from care homes not subsequently recruited to the pilot RCT. Feedback received from care home residents and staff, as well as experience gained by the research podiatrist informed the remodelling of the intervention and its delivery. Briefly, the process was as follows. Staff and residents participated in a 3-month acceptability-testing phase of the initial intervention, and feasibility test of the recruitment strategy. At the conclusion of testing, semi-structured interviews were conducted to assess the acceptability and perceptions of the intervention. Based on these findings, the intervention and its delivery were refined so as to be acceptable and feasible in the care home setting. Furthermore, the research podiatrist worked closely with the care home staff to develop suitable training in order to facilitate delivery of the intervention. Both the remodelling process and feasibility testing of the recruitment strategy are described in more detail elsewhere [23, 24].

### Control group

The control group received core podiatry only, which is defined as routine nail and callus (hard skin) maintenance provided either by the UK National Health Service (NHS) or podiatrists in private practice [30]. Within the geographical area in where the study was conducted (East of Scotland), the NHS provides core podiatry services routinely to all care home residents.

### Intervention group

In addition to the core podiatry that the control group received, the intervention group also received foot orthoses provision, footwear assessment and provision and a course of foot and ankle exercises.

#### Foot orthoses

Foot orthoses are reported to improve balance and postural stability by increasing tactile stimulation of the plantar surface of the foot, thereby enhancing afferent somatosensory feedback available to the central nervous system [37]*.* We supplied and fitted full-length, prefabricated foot orthoses (Formthotics™, Foot Science International, Christchurch, New Zealand). These are widely available in the UK and mainland Europe. The devices were dual density: the base layer is constructed of high (hard) density thermoformable closed cell polyethylene foam and the top layer constructed of low (soft) density thermoformable closed cell polyethylene foam. The devices were customised for each resident in two ways: firstly, by moulding the devices with a heat gun in order to match the contours of the foot, and secondly by affixing 6 mm thick Poron™ cushioning material (Rogers Corporation, Connecticut) under the forefoot area of the orthotic in order to redistribute pressure away from areas of high pressure (i.e. where plantar lesions such as hyperkeratosis were identified by research podiatrist (GW or ZY) on the plantar aspect of the foot. The cost of supplying orthoses for each resident was £24 ($A42; $31; €28). These orthoses were only supplied if the resident was not using orthoses that had been already supplied to them prior to their recruitment to the study.

#### Footwear assessment and provision

Residents’ current footwear was assessed by the research podiatrist using a validated footwear assessment tool [38]. In cases where residents’ footwear was judged inappropriate, residents, family members, and/or relevant care home staff were given a footwear catalogue containing a selection of footwear that, according to the validated footwear assessment tool, met the criteria for optimal footwear (DB Shoes, Northamptonshire, England). Residents were then able to select their preferred footwear from the catalogue, and these were then purchased on behalf of the residents and paid for from the study grant funds. The cost of footwear provision for each resident ranged from £75 to £89 ($A130 to $A154; $97 to $116; €87 to €102).

#### Foot and ankle exercises

Table 1 provides details of the exercise programme. The foot and ankle exercises were adapted from those conducted by Spink et al. [18] and Ribeiro et al. [39] to provide a regime that was acceptable within care home routines. The original exercise protocol, for example stated “3 repetitions of 10 sets”. Care home staff and residents found this to be potentially confusing, so this description would be changed to say simply, “30 times”. Participants were asked, in collaboration with the care home staff, to complete the exercise component of the intervention 3 times per week (on days of their own choosing) for 3 months. Exercise frequency remained at 3 times per week for the duration of the study. Only the ankle exercises involved progression.

**Table 1:** Exercise programme

Activity	Description	Frequency	Intensity progression*
Ankle dorsiflexion strength*	(i) Sit with knee extended. (ii) Wrap middle of exercise band around foot. (iii) Grasp ends of bands, and hold at waist, taking up slack. (iv) Push foot down into the band and return slowly.	3 × 10 repetitions	Increase tension strength of resistive exercise band
Ankle plantarflexion strength*	(i) Sit with both knees extended. (ii) Wrap middle of band around the foot of the ankle to be exercised. (iii) Run the band under opposite foot to hold band in place. (iv) Grasp ends of bands, and hold at knee height, taking up slack. (v) Lift foot against the band, hold and slowly return.	3 × 10 repetitions	Increase tension strength of resistive exercise band
Toe plantarflexion strength	(i) Pick up 25 mm diameter marbles and place in box	Pick up 2 × 20 marbles for each foot	None

*Participants began at an appropriate tension for their current strength capacity. This was determined by finding the tension at which 10 repetitions are possible with full range of motion before fatigue. Once the participant was able to perform 3 sets of 10 repetitions without fatigue, the intensity of the exercise was increased by increasing the resistance of the elastic band. Weekly visits from the research podiatrist allowed assessment of appropriate progression

#### Intervention training

Care home staff involved in the study participated in a 2-h training session conducted by the research podiatrists to familiarise themselves with the exercise component of the intervention. Intervention training to care home staff and residents was conducted by one of two podiatrists (GW or ZY). Information sheets detailing the exercises as well as a training DVD accessible via the study website, which was active for the duration of the trial (Additional file 1) supported staff in the exercise provision.

#### Intervention delivery

The intervention was administered in one of two ways: (i) if, on assessment, the resident demonstrated sufficient cognitive capacity and understanding they were, if they wished, trained to practice the exercises independently, or (ii) if residents did not have sufficient understanding either a family member or a member of care home staff was trained to supervise the exercise programme. Logbooks to record exercise adherence were issued to all participants and placed in a prominent area of each resident’s room.

### Sample size and statistical analysis

Since this was a pilot study, a formal sample size calculation was not conducted in advance [40]. We aimed to recruit 40 care home residents to the pilot trial, since it was felt that this would be sufficient to facilitate a later sample size calculation, whilst allowing for participant attrition. The primary falls-related outcome of interest was the number of falls. Cohen’s *d* [41] was also calculated to estimate the effect size of the intervention for the difference in mean falls between the groups at time points T1-T2, T2-T3, and T3-T4. We also used the negative binomial regression model to compare recurrent fall events in both groups [42]. T-tests for the difference in means were used to estimate *p*-values for between group differences in number of falls. Effect size was calculated for the difference in means in the secondary outcomes from T1-T2 and T3-T4. Adherence to the intervention was recorded via exercise logs that were completed either by the care home staff or the resident and analysed descriptively. Finally, median time to first fall was calculated as a safety measure to ensure that those in the intervention group were not at a higher risk of falling [43]. SPSS and Stata were used for the statistical analysis. Analyses were carried out using the intention to treat principle, by multiple imputation of missing at random data.

## Results

### Recruitment

**Fig. 1:**
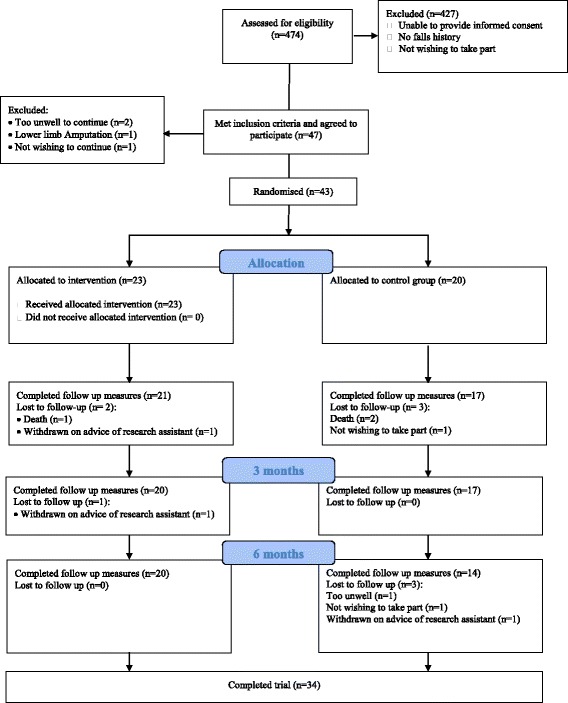
CONSORT flow of participants through study

Figure 1 shows the CONSORT [44] diagram with the flow of participants through the study. Between January 2014 and June 2014, we screened 474 potential participants from six care homes. The main reason for non-recruitment was inability to provide informed consent. Forty-seven residents (9.9%) met the inclusion criteria, agreed to participate and provided informed consent. Four residents dropped out prior to randomisation, thus 43 residents were randomised, representing 9.1% of the screened population. With the exception of gender, previous stroke, and eye problems, the intervention and control groups were well matched on the majority of baseline demographic characteristics (Table 2). Due to delays in the provision of personalised intervention equipment (orthoses and footwear), the mean number of days in the trial was longer in the intervention group: 257.9 (SD 57.8) compared to the control group: 208.1 (SD 86.5).

**Table 2:** Baseline participant characteristics

Characteristics at screening^a^	Podiatry intervention group *n* = 23	Usual care group *n* = 20	Total *n* = 43
Male, n (%)	3 (13%)	5 (25%)	8 (19%)
Female, n (%)	20 (87%)	15 (75%)	35 (81%)
Age (years), mean (sd)	86.9 (6.2)	85.9 (7.8)	86.4 (6.9)
Body mass index^b^, mean (sd)	24.2 (4.6)	28.3 (5.8)	26.1 (5.5)
MMSE, mean (sd)	21.2 (5.4)	21.0 (5.3)	21.2 (5.3)
Medical History, n (%)			
Diabetes	2 (8.7%)	2 (10%)	4 (9.3%)
Peripheral neuropathy	1 (4.3%)	0 (0%)	1 (2.3%)
Hypertension	12 (52.2%)	8 (40%)	20 (46.5%)
Previous CVA	8 (34.8)	3 (15%)	11 (25.6)
Peripheral vascular disease	1 (4.3%)	2 (10%)	3 (7%)
Angina	5 (21.7%)	3 (15%)	8 (18.6%)
Heart failure	2 (8.7%)	3 (15%)	5 (11.6)
≥4 prescribed medications	22 (95.7%)	19 (95%)	41 (95.3)
Eye problems (n, %)			
Partially sighted	4 (17.4%)	3 (15%)	7 (16.3%)
Registered blind	0 (0%)	4 (20%)	4 (9.3%)
Uses walking aids (n,%)	18 (78.3%)	18 (90%)	36 (83.7%)
Currently accessing podiatry services (n,%)	23 (100%)	20 (100%)	43 (100%)

*MMSE* Mini-Mental State Exam, *Higher score* better cognition

^a^For continuous variables, means and standard deviations are given. For categorical variables, proportions in each category are given

^b^weight/height^2^

### Retention

Between recruitment and randomisation, four residents dropped out. Reasons given: too unwell to continue (*n* = 2), lower limb amputation (*n* = 1), no longer wishing to take part (*n* = 1). Between T1 and T2, 4/23 (17%) participants in the intervention group dropped out, and 3/17 (18%) participants dropped out from the control group. Between T2 and T4 one further participant dropped out from the intervention group, and a further three dropped out from the control group. The final trial participant retention rate was 87% in the intervention group and 70% in the control group.

### Falls-related outcome measures

#### Number of falls

Table 3 shows the mean number of falls per participant and effect size by time point. 97 falls occurred from baseline (T1) to the end of the 6-month follow up period (T4), with 49 occurring in the intervention group, and 48 in the control group. Of all participants who started the trial, in the intervention group 48% (10/21) experienced at least one fall, and in the control group 12/17 (71%) experienced at least one fall. 7/21 (33%) participants in the intervention group had repeat falls, compared with 9/17 (53%) in the control group. There was one fall-related fracture in the intervention group (hip) and one fall related fracture in the control group (clavicle). Following multiple imputation to account for missing data on the nine residents who did not complete the trial, the podiatry intervention group experienced a total mean of 2.3 (SD3.2) falls from T1 to T4, compared to the control group who experienced a mean 2.7 (SD3.4) falls. The negative binomial model indicated no significant difference between the groups: incidence rate ratio 0.605, 95% CI 0.243 to 1.502, *p =* 0.3.

**Table 3:** Mean number (range) of falls per participant and effect size by time point

Outcome measure	Time point	Podiatry intervention group (*n* = 23)	Control group (*n* = 20)	*p*-value (95% CI)	Standardised effect size^a^ Cohen’s d (95% CI)
Mean (range) falls per participant	T1-T2	0.64 (0–3)	1.18 (0–6)	0.08 (−1.39 to .31)	0.4 (−0.2 to 1.0)
	T2-T3	0.99 (0–5)	1.05 (0–3)	0.18 (−0.88 to .82)	0.0 (−0.5 to 0.6)
	T3-T4	0.77 (0–7)	0.83 (0–6)	0.47 (−0.82 to 1.11)	0.0 (−0.5 to 0.6)

*T1* Baseline, *T2* Follow up at end of intervention, *T3* Follow up 3 months after end of intervention, *T4* Follow up 6 months after end of intervention

^a^Derived from the difference in means of the 2 groups; positive value favours intervention

The effect size from T1 to T2 appeared to favour the intervention on the number of falls (Cohen’s d = 0.4; 95% CI -0.2 to 0.1). This effect size was not maintained and reduced to no effect at T3 and T4.

#### Time to first fall

Participants allocated to the intervention had a median falls-free survival time (time to first fall) of 91 days (range 42 days to 257 days). This was longer than those in the control group who had a median time to first fall of 64 days (range 2 days to 160 days). Time to first fall did not differ significantly between the groups (Log Rank test [Mantel Cox] *X* ^2^(1) 0.67, *p* = 0.41). These data indicate that those in the intervention group were not placed at higher risk of experiencing a fall compared to the control group as a result of the intervention.

#### Secondary outcomes

Table 4 shows the means and effect sizes for all secondary outcomes in the domains of balance, functional ability, strength, quality of life, activities of daily living, falls self-efficacy, and foot problems.

**Table 4:** Means and effect sizes on secondary outcome measures, calculated from means of each outcome in the groups at T2 and T4

Measure	Podiatry intervention group (*n* = 23)				Control group (*n* = 20)				Standardised effect size^a^ Cohen’s d (95% CI)	
	T1	T2	T3	T4	T1	T2	T3	T4	T2	T4
Timed Up and Go Test^b^(seconds), Mean (SD)	43.1 (42.3)	67.8 (62.6)	69.3 (78.9)	93.0 (105.7)	48.1 (28.6)	66.0 (47.6)	60.1 (36.8)	61.8 (55.8)	0 (−0.6 to 0.5)	−0.4 (−0.9 to 0.2)
Ankle joint strength (Newtons)^c^, Mean (SD)										
Dorsiflexion	40.0 (12.5)	48.4 (18.8)	47.8 (12.5)	53.3 (12.8)	39.7 (17.1)	49.4 (14.2)	54.1 (9.9)	55.4 (12.6)	0 (−0.6 to 0.5)	−0.1 (−0.8 to 0.4)
Plantarflexion	63.4 (25.8)	73.7 (22.3)	75.0 (18.4)	80.2 (14.9)	63.8 (24.3)	88.8 (22.0)	84.1 (14.7)	87.7 (10.4)	−0.7 (−1.3 to −0.1)	−0.6 (−1.2 to 0.0)
Berg Balance Scale^d^, Mean (SD)	30.1 (13.2)	29.7 (13.9)	28.6 (14.1)	26.7 (13.7)	22.7 (12.1)	26.1 (12.4)	26.5 (12.0)	26.2 (11.1)	0.3 (−0.3 to 0.9)	0.0 (−0.5 to 0.6)
EQ5D UK Index Score^e^, Mean (SD)	0.7 (0.3)	0.6 (0.3)	0.6 (0.2)	0.6 (0.3)	0.5 (0.1)	0.4 (0.3)	0.6 (0.2)	0.6 (0.3)	0.6 (0.0 to 1.3)	0 (−0.6 to 0.6)
Barthel Index^f^, Mean (SD)	77.0 (16.1)	71.9 (20.8)	73.2 (19.3)	71.5 (19.0)	73.8 (12.3)	71.1 (16.4)	72.8 (15.2)	68.5 (17.2)	0.0 (−0.5 to 0.6)	0.2 (−0.4 to 0.8)
Nursing Home Falls Self-Efficacy Scale^g^, Mean (SD)	2.4 (0.7)	2.4 (0.1)	2.3 (0.9)	2.6 (0.9)	2.5 (0.8)	2.5 (0.8)	2.5 (0.1)	2.5 (0.9)	−0.2 (−0.8 to 0.4)	0.1 (−0.5 to 0.7)
Podiatry Objective Clinical Score^h^ Mean (SD)	2.6 (0.6)	2.9 (0.7)	2.7 (0.6)	2.6 (0.6)	2.6 (0.5)	2.8 (0.7)	3.0 (0.7)	2.8 (0.7)	−0.1 (−0.7 to 0.4)	0.3 (−0.3 to 0.9)

*T1* Baseline, *T2* Follow up at end of intervention, *T3* Follow up 3 months after end of intervention, *T4* Follow up 6 months after end of intervention

^a^Derived from the difference in means of the 2 groups; positive value favours intervention

^b^Higher time denotes poorer performance; >30 s indicates falls risk

^c^Mean left and right score for each participant; higher score = better strength

^d^Min = 0, Max = 56; higher score indicates better performance

^e^0 = worst possible health state, 1 = best possible health state

^f^0–20 = total dependency, 21–60 = severe dependency, 61–90 = moderate dependency, 91–99 = slight dependency

^g^Reverse-coded such that a higher score indicates less concern about falling

^h^2 = slight problems, 3 = moderate problems, 4 = severe problems, 5 = gross problem

#### Feasibility of outcome data collection

Collecting data on the number of falls was straightforward since all care homes kept a record of fall events as part of their normal documentation processes. Completion of the secondary outcomes was as follows: Podiatry Objective Clinical Score (100%), Berg Balance Scale (100%), Timed Up and Go Test (93.4%), Barthel Index (100%), EQ-5D (95.3%), Nursing Home Falls Self-Efficacy Scale (100%) and ankle strength (98.7%), 100% completion was achieved for the Barthel Index. This was because we employed proxy completion of the Barthel Index by care home staff, which would be regarded as normal practice for this measure. Similarly, full completion was achieved for the Podiatry Objective Clinical Score because this relied upon clinician report following examination of the feet. Completion rate of the EQ5D was 95.3%. The issue preventing full completion was a number of residents having difficulty in understanding how to complete the visual analogue subscale of the EQ5D. Timed Up and Go Test had a completion rate of 93.4%. Dynamometry to measure ankle strength had a completion rate of 98.7%. We obtained data on every item within the Berg Balance Scale, and the Nursing Home Falls Self-Efficacy scale – if residents were unable to complete a particular section they simply received the minimum score possible for that particular manoeuvre or domain, as per the guidance for these measures.

#### Adverse events and blinding

No adverse events related to the intervention were reported. The rater maintained a diary of visits throughout the follow up period, and reported 11 instances of unblinding.

#### Adherence

Documented exercise adherence (defined by the percentage of participants who reported completing the exercise programme three times per week) was low (35%). Insole adherence was 48%. Thirteen of the 23 residents in the intervention group were assessed as having sub-optimal footwear, and subsequently received the footwear component of the intervention. All reported wearing these all or most of the time.

## Discussion

The purpose of this study was to assess the feasibility and the potential efficacy of a podiatry intervention to reduce falls in care home residents. We have shown that conducting an RCT of a podiatry intervention to reduce falls in care homes is feasible with regard to recruitment, retention, and intervention delivery. Since this was a pilot trial with a small sample size [40], the effectiveness of the intervention cannot be determined from our results. However the data reported here support testing the intervention in a definitive full scale multicentre trial to test effectiveness.

There were some notable differences in baseline characteristics between the two groups. The majority of the participants were female, however this is representative of the general care home population [45]. Furthermore, there were differences in baseline characteristics in the domains of eye, problems and history of stroke. This is most likely due to our small sample, and in a large trial, we would not anticipate such differences. A small proportion of the screened population of residents (9.9%) were eligible to participate in this study because inclusion was restricted to those who were able to provide informed consent. It is well recognised that recruitment problems are compounded in care home research by multi-morbidity and cognitive impairment [20] and this was the experience of recruitment in the present study. However, despite the low recruitment rate, we exceeded our recruitment target, but needed to screen 474 care home residents to achieve this figure (target = 32; actually recruited = 43). Therefore we have shown that recruitment problems are not insurmountable. It is important to note that the proportion of screened residents who were eligible is essentially a point prevalence figure that met our criteria. It is possible that in a larger study conducted with a much longer recruitment window, a much higher proportion of the population would have met the criteria, and been recruited to the study. Our experience has taught us a number of factors related to care home staff that may have assisted recruitment. First, we relied upon the knowledge of staff to identify residents who they felt would be able to provide informed consent. Second, we reinforced the study as valuable and of importance to their residents. Third, we were sensitive to the care home culture, its organisation, and the level of disruption that the study may have caused.

There was a difference in retention rates between the two arms of the trial, with the control group demonstrating higher attrition. Death, declining health, and no desire to continue with the trial, were the main reasons for this attrition in the control group, and in a small sample such as ours, this difference is not unexpected, particularly given the challenges of retention in studies involving older adults [46]. Our retention rate is lower than pilot trials of falls interventions directed at community dwelling older people (92%) [47], and community dwelling older people with dementia (91%) [48] Moreover, our retention rate was lower than pilot trials in care homes not specifically directed at falls, for example mobility training interventions (97%) [49] and incontinence interventions (91%) [50]. However, it should be remembered that the follow up period in our study was considerably longer (6 months) than these studies. Our retention rate was similar to the rate reported in a recent feasibility RCT of a falls intervention in care homes [8] and those reported in a number of other full scale care trials of care home falls interventions [51, 52]. This is both encouraging and contrary to messages about challenging retention in care home studies [20]. Engaging care home staff to assist with recruitment is an approach we would endorse. Furthermore, to enhance recruitment for a full trial, we would consider alternative design approaches such as cluster randomisation; this approach was not taken in this pilot RCT because the larger sample size requirement and the inherent cost and workload implications of a clustering would have been more costly than a pilot RCT would merit [53].

Our data suggested an effect size that appeared to favour the intervention, but only directly after intervention delivery (T2); this effect disappeared (Cohen’s d = 0) at 6-month follow up (T4). These data suggest that if indeed there is any protective effect of the intervention, it reduces rapidly. For any sustained effect of the intervention, care home residents may need to persist with the intervention components on a regular basis. We do not know if, and to what extent, residents continued with the intervention after the end of the protocoled 3-month intervention period, although the reduction in effect size over time suggests that they may not. Our study suggested a trend towards longer falls free survival time in the intervention group compared to the control group, however this measure is not intended to be indicative of any potential benefit of the intervention, rather it was included as a safety measure to ensure that those receiving the intervention were not placed at a higher risk of experiencing a fall as a result of the intervention [28]. Although there are doubts about the validity of applying multiple imputation to missing data in small sample sizes, we felt it was appropriate to do so in this case because: 1) it is not acceptable to ignore missing values and the reason they arose [54]; 2) whether imputation is used or not, our statistical analysis needs to be treated with caution, 3) We considered this was the best way to get as accurate an estimate of potential effect as possible [54].

With regard to the secondary outcomes, the differences between the groups were varied, and in some cases contradictory. Despite apparent improvements in balance (Berg balance scale), performance in functional ability (Timed Up and Go Test) worsened both between and within the groups. A possible explanation for this could be declining overall health of our sample. However, the results in the secondary outcomes should be interpreted with caution since we conducted a large number of tests in a small sample. Therefore, as would be expected there was a high degree of variability. Furthermore, in our analyses we did not adjust for multiple testing and the probability that these findings were due to chance cannot be excluded. There were no other important differences between the groups on secondary outcomes and in a future definitive RCT, we would recommend a reduction in the number of secondary outcome measures. This would reduce the burden on participants, and focus interest on outcomes that are most meaningful for residents of care homes, i.e. falls-free mobility and quality of life [55].

Monitoring adherence to the exercise component of the intervention was a challenge. We recorded 35% of participants completing the exercise programme in full. As a result of low rates of diary completion it is difficult to determine to what extent participants adhered to the intervention. We would use the term, ‘*documented adherence’*, since the results (falls reduction at T2, and increased ankle joint muscle strength) would suggest that residents were completing the exercises more than they were actually documenting. This raises important issues about how adherence to interventions such as this is measured. To monitor adherence, PIRFECT relied upon self-report methods. This approach is fast, inexpensive and easy to implement. Conversely, this approach has poor reliability and validity because of response biases such as acquiescence, social factors such as perceived consequences, and psychological factors such as memory, cognitive ability, and health beliefs [56]. Accurately measuring adherence in exercise interventions is a problem that remains to be solved, although real-time data collection using hand-held mobile devices may hold promise [56]. In addition, the use of accelerometers may be considered in measuring adherence to exercises, however, their use is limited in large clinical groups because they are costly, and require the participant to consistently wear the device, and are not sensitive enough to assess individual limb exercise. [57].

The present study must be viewed in the context of its limitations. First, given the study’s low power and relatively short follow up time, the statistical analysis should be treated with caution. Secondly, because of the nature of this multifaceted intervention, it was impossible to blind participants to their group allocation, nor was it possible identify the contribution of each intervention component to reducing falls in the intervention group. Fourth, within care homes, residents who are unable to provide informed consent are the rule rather than the exception, and the fact that inclusion was restricted only to those who could provide informed consent limits the external validity of this study. However, in comparison with other care home falls studies, the mean MMSE score of our sample was 21.2 (classified as mild cognitive impairment), which is only slightly higher than a previous care home falls RCT by McMurdo [58] whose sample had a mean MMSE of 19, and lower than Jensen [52] whose sample had a mean score of 24. It should be remembered that whilst useful as an indicator of cognitive ability, the Mini Mental State Examination score may mislabel residents who can consent as lacking capacity, and residents unable to consent as having capacity [59]. Therefore, recruitment for a subsequent larger study will require ethical approval that allows for the inclusion of residents without capacity to consent, and it is important to consider how this may be achieved in a future definitive trial. Our study was carried out in Scotland where current laws do not allow for consent by proxy for those lacking capacity. However, in a future multicentre trial, as well as recruiting in Scotland, we would intend to recruit in sites in England, where consent by proxy is permissible in law. Lastly, whilst we undertook some process evaluation work during the development of the intervention, our approach did not adhere to published frameworks. The importance of process evaluations has long been recognised, and there is now published guidance on the practical steps for its conduct [60]. Therefore, we will incorporate a formal full process evaluation using this practical guidance in a future trial.

Intervention cost and cost effectiveness is an important consideration. Cost effectiveness could not be determined from a small pilot study, however we can make some remarks about the potential overall cost of the intervention. Purchase of the footwear and orthoses components of the intervention are likely to be the most expensive aspects. However, whoever bears this cost will be dependent upon the health service delivery context in which intervention is delivered. The cost of footwear could conceivably be borne by the resident in some cases. In terms of administering the intervention, apart from initial training from a podiatrist, the exercises are embedded into the normal care home routines and are delivered by care home staff, thus incurring minimal extra cost for health services. The footwear and orthoses aspects of the intervention can be delivered in two visits, with one visit for assessment, and one visit for fitting.

## Conclusion

Delivering and testing a podiatry intervention to reduce care home falls is feasible, despite the numerous challenges that care home research poses. The intervention and trial was also deliverable in care homes of differing sizes and models of ownership (state-owned, privately-owned). This pilot study not only provides essential information to enable the design and implementation of a full-scale RCT of the intervention, but is also likely to inform other future studies using complex intervention development frameworks with the care home population. The intervention itself is low-cost, easy to implement and uses skills that are standard to podiatrists internationally. Overall, our data support progression to a full multi-centre RCT that incorporates a full process evaluation.

## Additional file

Additional file 1: DVD media that was developed as part of the intervention delivery training programme supplied to the care home staff during the study. (M4 V 53690 kb)

## Abbreviations

BBS: Berg Balance Scale
EQ-5D: Euroqol five dimensions questionnaire
MMSE: Mini Mental State Exam
MRC: Medical Research Council
NHFSS: Nursing Home Falls Self-Efficacy Scale
NHS: National Health Service
POCS: Podiatry Objective Clinical Score
RCT: Randomised controlled trial
UK: United Kingdom
UKCRC: United Kingdom Clinical Research Collaboration
